# Bulk milk ELISA and the diagnosis of parasite infections in dairy herds: a review

**DOI:** 10.1186/2046-0481-66-14

**Published:** 2013-07-25

**Authors:** Mary Sekiya, Annetta Zintl, Michael L Doherty

**Affiliations:** 1 UCD School of Veterinary Medicine, University College Dublin, Belfield, Dublin 4, Ireland

**Keywords:** Bulk milk ELISA, Dairy herds, Parasite infections, Ostertagia, Fasciola, Dictyocaulus, Neospora

## Abstract

The bulk milk enzyme-linked immune sorbent assay (ELISA) is a rapid and inexpensive method of assessing herd exposure to pathogens that is increasingly being used for the diagnosis of parasite infections in dairy herds. In this paper, with the dairy herd health veterinarian in mind, we review the principles of the assay and the recent literature on the potential role of bulk milk ELISA for the diagnosis of ostertagiosis, fasciolosis, parasitic bronchitis due to cattle lung worm and neosporosis. It is generally accepted that assay results reflect exposure to the parasite rather than the presence of active infection. Bulk milk ELISA can be a useful tool for the veterinary practitioner as a component of a herd health monitoring programme or in the context of a herd health investigation. It can also play a role in regional or national surveillance programmes. However, the results need to be interpreted within the context of the herd-specific health management, the milk production pattern and the parasite life cycle.

## Introduction

This review paper emerged from discussions within the Animal Health Ireland (AHI) Technical Working Group for Parasite Control which identified a need to seek as much scientific clarity as possible in relation to the usefulness of bulk milk testing for parasite infections within the Irish dairy herd. AHI is an industry-led, not-for-profit partnership between livestock producers, processors, animal health advisers and government, with a remit encompassing diseases and conditions of rrlivestock that are endemic in Ireland but which are not currently subject to regulation [1]. Work programmes have been built on the animal health priority areas [2] including parasite control and biosecurity. At the core of each work programme is a Technical Working Group (TWG), or group of experts in the relevant fields. In keeping with the principle of maintaining standards of scientific excellence, the outputs of the working groups are subjected to peer-review and, where possible, published in international peer-reviewed journals.

The enzyme-linked immune sorbent assay (ELISA) is an immune assay which relies on the detection of host antibody as an indicator of infection. Once it has been developed for the analysis of individual serum samples it is frequently applied to individual and bulk milk analysis. In general terms, bulk milk ELISA is an attractive option for monitoring or establishing infection status in dairy herd health management as it provides an automated, rapid and relatively inexpensive method of assessing herd-level status with regard to various pathogens including Bovine Viral Diarrhoea Virus (BVDV), Infectious Bovine Rhinotracheitis (IBR), Salmonella and parasites [3-6].

### Underlying technology

ELISA development begins with the identification of parasite-specific antigens that elicit a strong immune response in the host. Once a suitable immunodominant protein antigen has been identified, it can be used to capture parasite-specific antibodies. The gene for the protein may also be cloned and expressed as a pure recombinant protein [7-9]. Recombinant proteins are uniform and can be produced in quantity but they generally represent only one or a few parasite proteins and lack post-translational modifications that may be important for their immunogenicity.

Most bulk milk assays use the indirect ELISA format. Antigen is coated on a microwell plate, the test sample, containing antibodies, is added and antibodies specific to the parasite bind to the antigen. A detection antibody, conjugated to an enzyme, commonly horseradish peroxidase (HRP) that catalyses the conversion of a substrate, results in a colour change (optical density) (Table 1) that can be measured using spectrophotometry. Negative samples result in a low optical density (OD) value due to failure to convert substrate and positive samples result in a quantifiable OD reading that is higher than the cut-off OD value (Table 1) [10].

**Table 1:** Explanation of the terminology used in this review

**Term**	**Explanation**
Gold standard	A perfect definitive test that produces no misclassifications
Sensitivity	Probability of a positive test result given the animal is truly infected
Specificity	Probability of a negative test result given the animal is truly not infected
Titre	The highest dilution of the sample at which the test is still positive
Optical density (OD)	Colour (absorbance) change in a sample resulting from the conversion of substrate and measured using spectrophotometry
Cut-off OD value	The absorbance above which samples are considered positive
Receiver operator characteristic	Statistical method used to calculate cut-off OD values
Sample to positive (SP) ratio or Optical density ratio (ODR)	The ratio of the OD of the sample to the OD of the positive control (SP ratio = OD_sample_/OD_positive control_)
Percent positivity (PP)	Sample to positive ratio x100

### Validation of the ELISA for bovine serum samples

ELISA assays are validated by comparing results with a ‘gold standard’ assay (Table 1), which provides indisputable evidence that an animal is infected with the parasite. A ‘gold standard’ might represent the parasitological detection of eggs, larvae or oocysts in a faecal sample or the verification of disease status by post-mortem examination. Results from the gold standard assay are compared with ELISA scores from the same individuals in order to determine suitable cut-off values that provide the highest possible sensitivity and specificity (Table 1). A statistical method that is commonly used for this purpose is the receiver operator characteristic (ROC) (Table 1) [11]. Originally developed to distinguish signal from noise in radio frequencies, the ROC provides a measure of how accurate a test is when compared to the gold standard. Suggest use Sensitivity probability of a positive test result given the animal is truly diseased and the specificity probability of a negative test result given the animal is truly non-diseased [12]. Alternatively, the cut-off value can be determined by testing a pool of known negatives. Suggested cut-offs are given as the mean OD of the known negative samples plus 2 or 3 standard deviations depending on the degree of stringency required [13]. The results from ELISAs are often reported as percent positivity (PP), sample to positive (SP) ratio or optical density ratio (ODR) (Table 1).

### Development of the ELISA for individual and bulk milk samples

In cows, immune responses to infection can be measured in milk as well as in sera. However, antibodies appear earlier in sera than in milk and the concentration of serum antibodies is approximately 30 times greater than in milk [14]. In milk, the predominant immunoglobulin is IgG1 (representing about 80% of the total immunoglobulin content), which is transported by active receptors on mammary alveolar cells [15]. Individual and bulk milk samples can both be tested by ELISA, however, there are significant differences in the interpretation of the results. Bulk milk samples are pooled samples and represent all lactating animals that contribute to the tank. There are many factors that can affect the titre (Table 1) of parasite-specific antibodies in the bulk milk including the number and relative seropositivity of contributors, stage of infection, stage of lactation, illness due to infection, and milk yield [16].

It is also important to note that a negative result from a bulk milk ELISA does not mean that the herd is definitively free of a particular parasitic infection. All ELISAs have a threshold antibody concentration that must be achieved before the bulk milk assay tests positive. Intuitively, one would assume that the lower the OD value for the bulk milk, the fewer infected animals are contributing antibodies to the pooled sample. However, correlating the percentages of infected animals with a bulk milk score can be challenging. This measurement is known as ‘within-herd prevalence’, and the minimum within herd prevalence gives an approximate threshold cut-off for a positive test result. There are several approaches to determining within-herd prevalence, the most common is to calculate the percent seropositivity of individual animals contributing to the bulk-tank pool, and to correlate this value with the bulk milk score applying regression analysis [17,18].

### Application of bulk milk ELISAs for the diagnosis of infection status and surveillance

In addition to the factors mentioned above, bulk milk ELISA may be further biased because it clearly does not include contributions from non-lactating animals, those withdrawn from the milking pool due to disease or those treated with substances that require milk withdrawal [16]. Finally, bulk milk ELISA is subject to the same shortcomings as individual serum ELISA because there can be significant delays between onset of an infection and detection of the antibody and/or a lag between the elimination of the parasite and corresponding reduction in antibody titre. These in turn are influenced by treatment, re-infection or host immune response and clearance of the parasite.

Nevertheless, bulk milk ELISA results can provide timely information about parasite exposure status within the larger picture of a herd health monitoring programme. Monitoring on a regular basis (approximately 4 times/year) may demonstrate trends of parasite-specific antibody levels and seasonal variations in disease status. Bulk milk ELISAs can also be useful tools for measuring the relative intensity or prevalence of parasite infection in the herd [19-21].

Vercruysse and Claerebout (2001) [22] reviewed a range of parasitological and immunological techniques used to detect common diseases of livestock in the context of their ability to diagnose clinical and subclinical disease. Three thresholds were proposed: (1) a therapeutic threshold, where animals exhibit clinical signs, (2) a production-based or economic threshold, where individuals in a herd harbour subclinical infections that affect productivity and (3) a preventive threshold that can be used to predict future infections to inform appropriate control measures. Results from bulk milk assays are effective in determining production-based thresholds since they provide a useful indicator of subclinical infections and the relative infection status of a herd [8,21,23].

### Stomach worm, *Ostertagia ostertagi*

#### Life cycle and clinical signs

The nematode, *O. ostertagi* is the most important parasite contributing to bovine parasitic gastroenteritis in temperate and subtropical regions [24]. Eggs shed by infected individuals onto pasture, hatch under suitable environmental temperatures (above 10°C, optimum 23-25°C) and continue to develop within the faecal pat. As rainfall causes the pat to break up, infective third stage larvae emerge onto the herbage. When a new host ingests the larvae, they moult in the rumen and then burrow into the abomasal gastric glands. Finally adult worms emerge into the lumen of the abomasum. The pathology caused by ostertagiosis is chiefly associated with the larval migratory activity which causes structural and functional changes to the gastric glands, resulting in loss of function and impairment of the digestive process. This is exacerbated by host immune and inflammatory responses to the parasite and its products. Heavy infections are characterised by profuse watery diarrhoea and anorexia resulting in significant loss of body weight and condition. Subclinical infections, on the other hand, have been associated with economic losses due to impaired performance and milk yield [25]. Type I ostertagiosis usually occurs in calves from mid-July and is associated with high morbidity but low mortality. In contrast, type II ostertagiosis is generally seen in yearlings in the subsequent winter or spring. In this case, infections result from the delayed maturation of larvae ingested during the previous autumn. While the numbers of individuals affected by type II ostertagiosis is generally small, mortality rates amongst these may be high unless effective and timely treatment is provided.

### ELISA assays for the detection of *O. ostertagi*

An ELISA originally developed for the detection of serum antibody against *O. ostertagi* was first applied to milk in 1993 [26]. Using adult worm extract as capture antigen, Kloosterman and colleagues noted a significant correlation between bovine serum, individual milk and bulk-tank milk antibody concentrations [26]. Since then Svanovir has developed a commercial product (available from Boehringer Ingelheim Svanova, Uppsala Sweden) which can be used to screen bulk milk samples. The antigen is crude worm extract and results are reported as ODR. The kit also provides a conversion chart (developed by Forbes and Charlier [19]) that links ODR with predicted loss in milk yield and can be used to estimate likely economic losses.

It is important to stress that the relationship between serum, individual milk and bulk milk samples can be complex. A study in Sweden reported that median ODR was less for bulk milk than for serum but greater than those measured for individual milk samples [27]. Assessing individual and bulk milk ELISA ODRs from two dairy herds in Normandy over a one year period, Charlier and co-workers [21] also found that bulk milk ODRs were higher than mean individual milk ODRs. The authors suggested that this may be due to a greater contribution to the bulk milk tank by individuals with high antibody titres. Use of the bulk milk ELISA is further complicated by the fact that the crude antigen assay may cross-react with other bovine helminths, such as *Cooperia oncophora* and *Fasciola hepatica*[28].

### Association of bulk milk *O. ostertagi* antibody levels with production parameters

While *O. ostertagi* is present on all farms, the impact of the parasite on production and the potential value of treatment can be estimated by the level of antibodies detected. A range of studies have confirmed that *Ostertagia* bulk milk antibody levels are negatively associated with milk yield [25,27,29-32]. In addition there may be a small but significant decrease in milk protein content. Bulk milk ELISA scores increased with age of cow and the number of lactations [25,30,31] reflecting higher levels of specific antibody in older cattle [24]. Furthermore, the age at first calving was positively associated with bulk milk antibody levels (expressed as ODR) and Holstein herds had higher ODRs compared with Normande or Montbeliard herds [21].

Significant research effort has gone into the development of the *Ostertagia* bulk milk ELISA as a quantitative test that can be used to predict likely production losses associated with elevated antibody levels in the bulk milk tank. One of the most comprehensive studies by Forbes and colleagues [29] has given rise to the chart mentioned above, which is used in the interpretation of ODR scores for the Svanova *Ostertagia* ELISA kit [19].

### Effects of management practices on bulk milk *O. ostertagi* antibody levels

As would be expected from the epidemiology of the parasite, the most important management factor affecting antibody levels in the bulk milk tank is the extent to which animals have outdoor access to pasture [19,29,30,32-35]. No access to pasture resulted in low antibody concentrations, while in animals kept outdoors, antibody levels increased with the level of access to fully grassed pasture and herbage. There was also a proportional increase of bulk milk antibody level (measured as ODR) with percent of time spent grazing daily. Herds that were managed by summer grazing and winter housing demonstrated a seasonal pattern of high ODR in late summer and early autumn and low ODR in winter [31,33] reflecting the build-up of parasite larvae on pasture in mid-summer [24]. Furthermore, bulk milk ELISA scores increased the earlier the date of turnout and the later the month of housing [28,29]. Extensive production systems and organic herds with smaller herd sizes and lower stocking densities tend to have higher bulk milk antibody levels than animals in intensively managed systems [28,29,32,36].

Anthelmintic treatment of either the entire herd or milking cows at calving causes a decline in bulk milk ELISA scores [35], however, not all animals in the herd respond to the same degree. Sanchez and co-workers found that highly positive cows showed a greater response to treatment as measured by milk yield [37], than cows with lower levels of milk antibody and recommended using individual, rather than bulk milk testing to predict the milk production response after anthelmintic treatment [38].

While certain climatic variables such as rainfall, temperature and vegetative index also affect bulk milk antibody levels, it is thought that, within a given biome, management practices have a higher potential impact than environmental factors [35]. Prevalence of *Ostertagia* is significantly higher in central European countries than in Scandinavian countries as shown in Table 2, which may indicate a role of climate in the parasite success.

**Table 2:** ***Ostertagia ostertagi *prevalence based on bulk milk assay**

**Country (region)**	**Number of herds**	**Prevalence**	**Reference**
Belgium	Conventional 1,800	59.1% (95% CI, 56.8-61.4%)	[39]
Sweden	Organic 113	0.82% (95% CI, 0.78-0.86%)	[36]
	Conventional 113	0.66% (95% CI, 0.61-0.71%)	

### Prevalence of *O. ostertagi* according to bulk milk ELISA

According to two large scale bulk milk surveys, *Ostertagia* prevalence in Ireland/UK is intermediate-to-high compared with other European countries (Table 3) [29]. It is thought that this is largely due to the high proportion of grass in the cows’ diet (42% of herds were fed exclusively on grass, compared with Germany, where grass comprises less than 50% of the diet of most of the herd). In addition, Ireland with its temperate climate has the longest average grazing season at 7.4 months, (grazing was shortest for Sweden at 4.5 months, with the other countries intermediate).

**Table 3:** **Mean optical density ratios (ODR) for *Ostertagia ostertagi *based on bulk milk assay**

**Country**	**Number of herds pastured/total**	**Mean ODR**	**Reference**
Denmark	146/146	0.48	[29]
Germany	78/131	0.48	[29]
Italy	47/140	0.31	[29]
Netherlands	243/288	0.45	[29]
Portugal	92/163	0.61	[29]
Spain	91/143	0.53	[29]
UK/Ireland	142/174	0.60	[29]

### Liver fluke, *Fasciola hepatica*

#### Life cycle and clinical signs

The liver fluke or *Fasciola hepatica* is found worldwide in temperate regions and has a complex life cycle that is dependent on an intermediate snail host. Eggs that are passed in the faeces of an infected final host, develop and release motile ciliated miracidia onto the pasture. When the parasite encounters its intermediate host, the amphibious snail *Galba truncatula*, it penetrates via the integument and develops through the sporocyst and redial stages to the cercaria stage, which is shed by the snail. Following attachment to the vegetation, the cercariae encyst into infective metacercariae. When the final mammalian host ingests metacercariae, they excyst in the small intestine, migrate through the gut wall, and, after crossing the peritoneum, penetrate the liver capsule. Juvenile flukes burrow through the liver parenchyma for 6–8 weeks, then enter the bile ducts (occasionally also the gallbladder) where they reach sexual maturity [24]. Clinical signs resulting from heavy parasite burdens are characterised by anaemia, damage to liver parenchyma and submandibular oedema (‘bottle jaw’). In cattle, in contrast to sheep, acute disease only occurs occasionally, mostly in young calves following heavy challenge. Chronic infections, on the other hand, are common, causing reduced milk yield and quality [24].

### ELISA assays for the detection of *F. hepatica*

Several ELISAs have been developed for the detection of *F. hepatica* infection in bulk milk samples, these include the Idexx ELISA serum and milk kit (formerly the Institut Pourquier ELISA - Idexx, Westbrook Maine, USA) [40], the MM3-Sero ELISA, an ELISA based on a monoclonal antibody, that is used to capture specific *F. hepatica* ‘excretory-secretory’ (ES) antigens [41] and the University College Dublin (UCD) assay which relies on a recombinant mutant Cathepsin L1, the immunodominant protein found in ES [9]. However, the most widely used ELISA in published studies is an in-house assay developed at the Liverpool School of Tropical Medicine that uses the ES fraction of the parasite as capture antigen [17]. ES antigens are immune modulatory molecules actively shed from the surface of helminth parasites or released through specialised excretory or secretory organs [42]. Table 4 summarises the different available ELISA formats for bulk milk testing. All kits also have a high sensitivity and specificity for individual sera and milk. The minimum within-herd prevalence levels range from a low of 12% for the MM3-SERO ELISA to approximately 25% for the ES-ELISA.

**Table 4:** **Performance characteristics and minimum within-herd prevalence for four *Fasciola hepatica *ELISA kits**

**Kit**	**Antigen**	**Sensitivity (individual Sera/milk)**	**Specificity (individual Sera/milk)**	**Minimum within herd prevalence**	**Supplier**	**Reference**
IDEXX- Institut Pourquier	Fraction f2 of ES	95%^a^	98.2%^a^	20%^b^	IDEXX	^a^[43]
						^b^[16]
MM3-SERO	Monoclonal Ab sens. wells treated with purified protein	100%^c^	100%^c^	12%^d^	Bio-X	^c^[44]
						^d^[41]
LSTM ES-ELISA	ES fraction	98% (95%CI 96–100%)^e^	96% (95% CI 93–98%)^e^	25%^f^	In-house	^e^[17]
						^f^[45]

### Association of bulk milk *F. hepatica* antibody levels with production parameters

While most studies agree that elevated *F. hepatica* antibody levels in bulk-tank milk samples are associated with decreased milk yield [46,47], a reduction in milk solids or fat content has been reported by some workers [46,48]. In addition, herds with higher antibody levels tend to have longer intercalving intervals, reflecting the potentially negative effects of liver fluke infections on conception and pregnancy rates [49]. It is likely that many of these effects are only detectable when comparing highly positive to negative herds [47], indicating that the magnitude of the parasite burden may be fundamental.

In addition to affecting production parameters and thus causing economic losses, *F. hepatica* has been implicated as an immunosuppressive agent. More specifically, the fluke is thought to increase susceptibility to certain bacterial infections and may inhibit the inflammatory response to the intradermal tuberculin test [50].

Reichel and co-workers stated that the issue of the duration of the antibody response in relation to recently treated infections remained unresolved and the persistence of antibodies after treatment could lead to ‘false positives’ [18]. This point serves to highlight the importance of adopting an overall herd health approach with attention being paid to the cows in the context of clinical and subclinical disease as well as to other diagnostic tests including coprological examination.

### Effects of management practices on bulk milk *F. hepatica* antibody levels

Generally fluke infections cluster in areas where environmental conditions are suitable for the larval life cycle stages and the intermediate host, the mud snail, *G. truncatula*[39]. However, using bulk milk ELISA screening as an indicator for economically significant liver fluke burdens, Bennema and colleagues found that in addition to climatic and environmental factors, herd management practices had a major impact [51]. Bulk milk ELISA scores increased with the proportion of fresh grass in the diet and the length of the grazing season, both factors that are directly linked to the exposure to metacercariae, particularly in the autumn when infection levels on pastures peak. Finally, and rather suprisingly, medium-sized herds (30–60 animals) were more likely to be bulk-milk positive than large-sized herds (>60). However, this was thought to be due to confounding, underlying management factors, not addressed in the study.

### Prevalence of *F. hepatica* according to bulk milk ELISA

Bulk milk screening indicated high prevelances of between 50 and 85% of herds in the UK, Austria and Germany, with intermediate levels in Belgium and low prevalences in both conventional and organic farms in Sweden (Table 5). In Ireland, liver fluke has long been understood to be endemic. A study carried out in 2006 reported the presence of liver flukes in 65% of livers from culled cattle in Ireland [52].

**Table 5:** ***F. hepatica *prevalence based on bulk milk assay and in comparison to faecal analysis (where available)**

**Country/region**	Number of herds *	**Herd prevalence - Bulk milk**	**Herd prevalence - Faecal analysis**	**Reference**
England	623	48%		[45]
Wales	445	86%		
England	60	53%	17% (of pooled samples examined by standard sedimentation)	[53]
Belgium	1,800	37% (95%CI: 35-40%)		[39]
Sweden	113 (organic)	7%		[36]
	113	6%		
Austria	31	58% (Euroclone ELISA)	65% by sedimentation	[16]
		61% (Pourquier ELISA)	55% by coproantigen ELISA	
Germany	4630	51%		[54]

* All herds under conventional management unless otherwise indicated.

### Lungworm, *Dictyocaulus viviparous*

#### Life cycle and clinical signs

Like *O. ostertagi*, the cattle lungworm, *Dictyocaulus viviparus*, is a nematode of the trichostrongylid family with a worldwide distribution, although it is most common in temperate regions with high rainfall [24]. The adult female worms are ovo-viviparous and as a result larvae are present in fresh faeces, a feature that is highly unusual in gastrointestinal worms [24]. The migration of the larvae out of the faecal pat and into the herbage is aided by the fungus *Pilobolus,* which can propel the tiny parasitic larvae over a distance of up to 3m. Following ingestion, the parasites burrow through the intestinal mucosa and travel via the lymph or blood to the lungs, where they break out of the capillaries into the alveolar spaces. After some further maturation in the bronchioles, the adult lungworms appear in the bronchi. Clinical signs can appear some time before infections become patent (and detectable by faecal analysis). Dictyocaulosis is also known as parasitic bronchitis, and heavy infections are characterised by frequent bouts of coughing and dyspnoea due to widespread lung damage. In endemic areas most animals acquire protective immunity during their first grazing season and as a result, severe clinical signs are usually only observed in very young calves exposed to heavy challenge [24]. In older animals, subclinical or mild to moderate infections are common, and although the level of infection in endemic countries may be high, the number of animals that go on to become clinically affected is lower than those identified as seropositive. In a study on first season grazing cattle herds in northern Germany, it was estimated that infection with the parasite caused clinical disease in approximately one-third of infected cattle [55].

### ELISA assays for the detection of *D. viviparus*

The standard ELISA assay for the detection of *D. viviparus* uses as capture antigen a recombinant major sperm protein (MSP), which is the most immunogenic *D. viviparus* protein identified so far [56,57]. For individual serum and milk samples the recombinant MSP ELISA has a sensitivity of between 97.5 and 99% and a specificity of over 99%. Significantly, there is no cross reactivity with *Ostertagia* or *Cooperia*[8,13,58]. Experimental infections indicated that lungworm-specific antibodies were detectable 28 to 35 days post infection (dpi) for a period of between 79 and 107 days [58]. In animals turned out to pasture, ELISA readings exceeded cut-off values at 28 days post turnout. Generally antibody patterns in individual milk samples closely match those in individual serum samples but titres are lower.

For bulk milk samples the MSP ELISA is a useful tool only if the herd is highly infected (during moderate to severe outbreaks) [59]. According to a study of thirty-three farms in the Netherlands, a region with a historically high prevalence of lungworm infection, at least 30% of the animals in the herd were required to be seropositive before the bulk milk sample exceeded the cut-off.

### Association of bulk milk *D. viviparus* antibody levels with production parameters

The correlation between raised antibody levels according to bulk milk ELISA testing and lungworm infection status of the herd is not well understood. Ploeger and colleagues reported that bulk-tank milk antibody levels reflected the proportion of the herd that showed clinical signs such as coughing and increased respiratory rate [59]. However, bulk milk ELISA results mostly became positive *after* the onset of disease in the herd and were more closely related to incidence of lungworm-related morbidity than to prevalence of lungworm infection. The authors suggested that this might be due to the fact that the MSP antigen is a protein that is only expressed in the adult stages of the worm. Those authors concluded that the bulk milk ELISA had a role in the investigation of outbreaks of respiratory disease in adult cattle but that further research was needed before it could be routinely used as a monitoring tool in the context of disease prevention.

Recovery from dictyocaulosis can take several weeks to months [24]. During this time animals continue to suffer clinical signs, largely as a result of a persistent inflammatory response to the presence of dead worm material, damaged host tissue and, frequently, secondary bacterial infections. Even fully recovered cattle often show stunted growth. Generally recovered animals are immune to reinfection but exposure to massive larval challenge can result in the so-called ‘reinfection syndrome’. In this case migrating lungworm larvae stimulate a strong immune response that causes their destruction within the bronchioles before they can mature to adult worms. Resulting bronchiolar obstruction and the formation of lymphoid nodules around the dead larvae cause frequent coughing, tachypnoea and reduction in milk yield. If and in what way bulk milk ELISA can identify mild or subclinical infections has not yet been established. As adult D. viviparous stages are absent in animals with reinfection syndrome, these are not detected using the current MSP ELISA. For this, further research into lungworm antigen, particularly early larval stage antigen, is needed to provide alternative assays.

### Effects of management practices on bulk milk *D. viviparus* antibody levels

*D. viviparus* resembles *O. ostertagi* in its transmission route and seasonality, characterised by a gradual build-up of infective larvae on pasture over the summer months, and a general die-back during the winter (although some larvae may survive overwinter by migrating down into the soil) [24]. Hence, similar to ostertagiosis, access to pasture, particularly during times of greatest infection pressure, would be expected to be the most important factor affecting bulk milk antibody levels for lungworm. Unfortunately, there are no published studies on the effects of management strategies on *D. viviparus* on bulk milk ELISA scores. A surveillance study in Sweden reported a higher bulk milk prevalence of *D. viviparus* antibody in organic as compared to conventional dairy farms [36]. However, under Swedish animal welfare legislation all cattle over 6 months of age must have outdoor access for 2 to 4 months during the grazing season, and it is not known whether organically reared animals in the study did in fact spend more time grazing. According to the authors, the main difference between organic and conventional production systems in Sweden is that the prophylactic use of anthelmintics is prohibited in organic herds.

Because *D. viviparus* elicits a strong adaptive immune response in previously exposed animals, it is generally only calves in their first grazing season that are clinically affected [24]. However, some anthelmintic control strategies used in calves today are thought to be so efficient that many animals remain free from infection until they return to pasture during their second year as heifers. At this point they often suffer clinical disease because due to the lack of antigenic exposure they failed to develop effective immunity in the previous year [59]. It is likely, therefore, that anthelmintic use, particularly prophylaxis, would have a significant effect on antibody levels.

### Prevalence of *D. viviparus* according to bulk milk ELISA

The only data available for bulk milk prevalence in Ireland, were collected during an as yet unpublished study carried out in 2009 and 2011 which indicated a herd prevalence of 7% (Bloemhoff and Sayers, comm.). An abattoir study in Co. Kildare in 2002/2003 revealed first stage larvae in the rectal contents of 14% of culled dairy and beef cattle [55]. Thus the prevalence in Ireland is similar but perhaps slightly lower than that seen in central Europe (Table 6).

**Table 6:** ***Dictyocaulus vivaparus *prevalence based on bulk milk assay**

**Country (region)**	**Number of herds**	**Prevalence**	**Reference**
Belgium	1,800	19.6%	[39]
Sweden	Organic herds 113	18%	[36]
	Conventional 113	9%	
Germany (East Frisia)	906	12.8% Jan 07	[60]
		6.9% Sept 08	
		6.6% Nov 08	

### *Neospora caninum*

#### Life cycle and clinical signs

Only discovered in 1988, the protozoan parasite *Neospora caninum* is now known as a major cause of abortion in cattle worldwide [61,62]. The dog is the final host and can pass infective oocytes in its faeces from 8–23 days post infection [24]. Cattle become infected by ingesting contaminated feed, water or herbage (exogenous transmission). Infections in adult cattle have little clinical effect, however, in the developing foetus they can cause severe pathology. In pregnant cows the parasites can invade the uterus, where they multiply (as tachyzoites) causing focal lesions at the maternofoetal interface (endogenous transmission). If this occurs early in pregnancy, it is likely to result in mummification and abortion of the foetus. Later on in gestation, calves may be born underweight with severe neurological signs. However, in many cases, calves born to cows infected at a late stage in pregnancy are clinically normal but persistently infected. Parasites in these congenitally infected cattle can recrudesce when they themselves become pregnant, again with potentially lethal effects to the foetus.

Unfortunately, the factors that determine whether a previously infected cow will abort, or will give birth to a sick or healthy calf are poorly understood [63]. Abortion storms, the most dramatic manifestation of neosporosis, when more than 10% of the cows in a herd abort within a 12 week period, are thought to be caused by exogenous transmission arising from infected dogs (mostly pups) recently introduced to the farm. However, as the incidence of oocyst shedding in dogs is very low, this is a rare occurrence. The most common route of transmission in cattle is by the vertical route from dam to calf (endogenous), resulting in persistently infected calves [64]. Through its effects on fertility, *N. caninum* is thought to reduce milk production in adult dairy cows [24].

### ELISA assays for the detection of *N. caninum*

There are several commercial *Neospora* ELISA tests that have been validated for bulk milk testing (Table 7). Most of these assays use whole tachyzoite antigen as capture antigen. The notable exception to this is the BioK 192/5 from Jemelle (Belgium), which uses a recombinant protein of the major immunodominant tachyzoite surface antigen. Tachyzoites are the rapidly dividing stages of the parasites that, during the acute phase of the infection, invade the placenta and developing foetus.

**Table 7:** **ELISA assays for the detection of *N. caninum *in cattle bulk milk samples**

**ELISA assay**	**Capture antigen**	**Sensitivity (95% confidence interval)**	**Specificity (95% confidence interval)**	**Reference**
ISCOM ELISA (Boehringer Ingelheim Svanova, Uppsala Sweden)	Tachyzoite antigen mixed with iscoms ^1^	50%	81%	[65]
		(21-79%)	(72-89%)	
IDEXX *Neospora* antibody test	Whole sonicated tachyzoites	61%	92%	[66]
		(49-73%)	(87-98%)	
LSI ELISA (Lissieu, France)	Whole tachyzoite crude antigen lysate	47%	94%	[66]
		(35-60%)	(90-99%)	
Mastazyme® ELISA (Mast Diagnostics UK)	Whole tachyzoites	61-78%	75-96%	[67]
BioK 192/5, Jemelle, Belgium	Recombinant NcSRS2 protein	95%	96%	[7]

^1^ Immunostimulatory complex composed of quillaja saponin, cholesterol and phospholipids.

Most studies indicated a strong correlation between individual seroprevalences and bulk milk results [6,64-66,68], except that higher milk ELISA results are usually found at later stages of lactation as compared with the serum ELISA [69]. Generally about 10 to 15% of the animals in a herd must be seropositive for the bulk milk result to exceed the cut-off [23,66,68]. However, some workers found that bulk milk testing under reported prevalences [70,71]. As with other bulk milk assays, antibody levels in the bulk milk tank are not only dependent on the proportion of infected cows but also their antibody levels, lactation stage and milk yield [66,72]. These variables are likely to be more significant in small herds, where the introduction of one or two highly seropositive animals could convert the bulk milk sample. On the other hand, if most individual antibody levels are only just above the cut-off, bulk milk results might be negative even if more than 15% of animals are infected. In spite of these drawbacks, bulk milk ELISA testing is considered an effective tool in tracking *N. caninum* prevalence at herd level [68], particularly since control measures for the disease currently focus on minimising the seroprevalence within herds [23].

### Association of bulk milk *N. caninum* antibody levels with production parameters

The effects of *N. caninum* infection on milk yield are not clear-cut. While some studies report reduced milk production in seropositive cows, others observed no association between milk yield and individual serostatus (reviewed in [47]). At herd level, a negative association has been reported between average milk production and ELISA values for bulk-tank milk, with an average loss of 1.6 kg/cow/day in highly positive herds compared to seronegative or low positive herds [47]. Furthermore, risk of abortion in seropositive cows is between 2 and 26 times higher than in seronegative cows [64,73-76]. Significantly, this correlation was also observed in relation to bulk milk: a study of over 3200 herds in the German state of Rhineland-Palatine reported that the annual rate of abortion was 3% higher in farms that were bulk milk positive than in negative farms [69]. This strongly indicates that knowledge of the levels of exposure and herd history on *N. caninum* may inform prediction of abortion risk, however, this may be most relevant in regions with a very high prevalence of *N. caninum*[23].

### Effects of management practices on bulk milk *N. caninum* antibody levels

The number of dogs on the farm and dog density in the surrounding area have been identified as the most significant risk factors for bulk milk prevalence [77]. At the same time, it must be remembered that the most common route of transmission in cattle is transplacental transmission from dam to calf. Since no effective treatment is available to prevent either abortion or transplacental transmission, the only management practice open to the farmer is not to breed from seropositive animals. It is to be expected, therefore, that selective breeding together with restricting canine access would, over time, lead to a reduction in antibody levels in the bulk milk sample of a herd, but to our knowledge there are no published records.

### Prevalence of *N. caninum* according to bulk milk ELISA

Most of what we know about the prevalence of *N. caninum*-induced abortions in Ireland is gleaned from clinical pathology findings. According to the All Ireland Animal Surveillance Disease Report, 2011, 5.3% of aborted foetuses in the Republic and 7.7% in Northern Ireland tested positive for *N. caninum* either serologically, by histopathology or immunohistochemistry [78]. Earlier surveys from the Regional Veterinary Lab in Kilkenny reported that 7% of foetuses and 14% of recently aborted cows submitted for abortion between 1999 and 2003 were serologically positive for *N. caninum*[79]. These figures are significantly lower than those reported for the UK in general where 27% of diagnosed abortions were attributable to *N. caninum*[80]. Unfortunately, no published reports are available regarding *N. caninum* prevalence as determined by bulk milk assay. However, in-house testing on behalf of herd owners indicate that the prevalence is approximately 9%, (based on 2,200 bulk milk samples tested in three rounds) in 2011 and 2012 (Sekiya M., unpublished). Table 8 lists prevalences worldwide and indicates that rates are highest in warmer climates. Further surveillance and monitoring may lead to models associating climate with levels of *Neospora* occurrence.

**Table 8:** ***Neospora caninum *prevalence based on bulk milk assay**

**Country (region)**	**Number of herds**	**Prevalence**	**Reference**
Thailand (North and Northeast)	220	46%	[72]
Sweden	2,978	8.3% (95% CI, 7.3–9.3%)	[70]
Norway	1,657	0.7% (95% CI, 0.3–1.2%)	[81]
Canada	235- May 04	6.4%	[68]
	189- May 05	10.1%	
	235- June 05	10.2%	
Australia (South)	122	2.5% (95% CI, 1.4–3.6%)	[71]
Spain (Galicia)	276	56%	[23]

## Conclusions and future prospects

**Figure 1:**
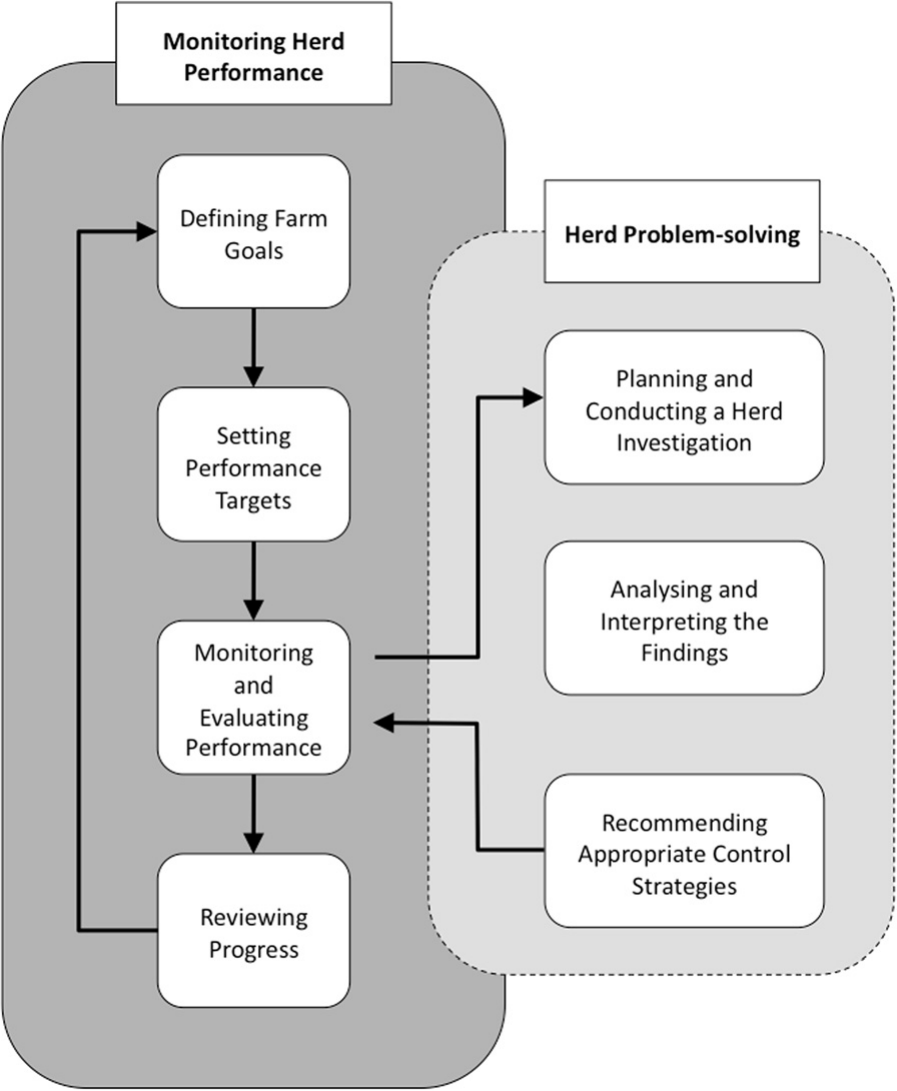
**The herd health management cycle, adapted from Mulligan et al. **[82]**.**

Dairy herd health management involves establishing and maintaining optimal animal health and productivity. The basic steps in delivery and execution of herd health management are cyclical. Initially farm goals and targets are defined, then herd performance in key areas is monitored and compared to agreed targets. Where shortfalls are identified, investigative protocols are employed to identify the cause and appropriate control strategies implemented. The effects of these controls on farm performance are monitored and thus the cycle begins again (Figure 1) [82]. This concept is central to all aspects of herd health management including parasite control. A dairy herd parasite control programme must be tailored for the individual farm taking animal health and production, farm-specific management, grazing history and seasonal conditions into consideration.

It is clear that bulk milk testing has a potential role in both the monitoring and investigative aspects of the herd health management cycle (Figure 1). However, its role needs to be seen in the context of the other key components of optimal parasite management in the dairy herd such as those outlined by the parasite control TWG/AHI [83].

Thus, the data from regular (at least 3–4 times/year depending on the calving pattern) bulk milk screening needs to assessed in the context of the other key components of parasite control including risk-based assessment of pasture contamination, judicious use of faecal testing as well as follow-up inspection of tissue (liver, lung, abomasum etc.) at post-mortem examination as well as in the context of abattoir surveillance. The bulk milk data could be viewed as one of the tools in the kit of the dairy herd veterinarian to facilitate decision-making at farm level.

Ostertagiosis makes its greatest economic impact (clinical and subclinical disease) in the context of first and second-grazing season calves and the decision to treat adult cows to improve milk yield must always be based on a proper cost-benefit analysis, whilst taking issues of anthelmintic resistance into consideration [84]. A bulk milk test for *O. ostertagi* antibodies at the end of a grazing season in the adult herd may assist the planning of worm control strategies for replacement heifers in the next season [85]. Thus, a test with a low titre at the end of the grazing season in the adult herd may indicate that exposure of first-grazing season animals that year was not sufficient to stimulate adequate immunity going into the second season.

Bulk milk monitoring is used to detect infections that are subclinical, yet result in increased costs to the herd owner primarily in terms of decreased milk yield and potentially, to a lesser extent, cattle weight gain, milk quality and reproductive fitness [22]. It is an effective diagnostic indicator of exposure to moderate to high levels of parasitic infections and can provide an indication of intensity of infection in the herd in endemic situations [23,33]. Finally, it has been investigated as an indicator for the effectiveness of parasiticide prophylaxis or treatment [22], bearing in mind, however, that antibody titres can remain high after treatment and that in these situations information provided by bulk milk testing needs to be considered carefully. As such bulk milk testing can inform cost-benefit analysis and treatment decisions. An added advantage of bulk milk testing is that the same samples that are already routinely collected by the dairy industry for milk quality testing can be used.

The application of bulk milk ELISA as a predictive tool for risks associated with parasite infection is still at an early stage, the extent of research findings varies with the parasite species in question. For *O. ostertagi*, liver fluke and lungworm, the risk of acquiring the parasite is linked to grazing on contaminated pasture. Bulk milk assay will give a good indication of current exposure if employed as part of an ongoing herd health surveillance programme. On the other hand, data from less frequent testing may be difficult to interpret as anti-parasite antibodies can persist for a long time post treatment (depending on assay). Available prevalence data from Ireland indicate that any herd on pasture is at risk of acquiring infection. The question then becomes: How severe is the herd level infection?

For *O. ostertagi*, bulk milk assay can be effective in providing thresholds that may be converted to predicted milk loss per cow per day [19]. For liver fluke, the risk is highly dependent on the environment and is linked to grazing on contaminated pasture. For lungworm, there is an advantage of knowing levels of exposure and how this might contribute to respiratory disease incidence. For *Neospora*, high levels of bulk milk antibodies may contribute to greater risk of abortion [23] and would indicate that *Neospora* should be considered as a cause in unusual patterns of abortion.

Bulk milk results contribute to building risk assessment models. An active area of research is the development of software models for the risk for infection and disease spread with a GIS based system, using prevalence data based on bulk milk assay in combination with other environmental factors including weather data and soil conditions [28,86,87]. One such programme is ParaCalc®, a spread-sheet model that calculates the effects of infections on production and the cost of the production losses, based on diagnostic assays of herd health and anthelmintic usage. The programme was tested during a study of Belgian dairy herds [88]. The results indicated an estimated median cost of infection with gastrointestinal nematodes of €46 per cow per year, with a much lower estimated cost of €6 for liver fluke. The most significant factor was reduced milk production in infected cows.

Integration of bulk milk assay results and other clinical findings in an easy to use application would be a tremendous advantage for both farmers and herd health management professionals. Future developments in bulk milk assay will likely include multiplexing platforms that facilitate the assay of several parasitic infections at one time and point-of-care or pen-side tests that provide an immediate result for the herd.

The bulk milk ELISA can be a useful tool for the veterinary practitioner as a component of a herd health monitoring programme or in the context of a herd health investigation. However, the results of bulk milk testing for gutworm, liver fluke and lungworm simple indicate the presence (or absence) of antibodies from prior or current exposure and do not necessarily indicate active infection or disease. Therefore, like all diagnostic tests, antibodies in bulk milk should be assessed with reference to the holistic herd health picture and not used as the only discriminator in the decision-making process with regards to both potential economic losses and response to treatment.

## Competing interests

The authors declare that they have no competing interests.

## Authors’ contributions

MD conceived the idea for the review and provided the herd health narrative. AZ provided the content relating to parasitology. MS provided the content relating to the diagnostic techniques. All authors read and approved the final manuscript.
